# Theoretical Investigation of Twist-Angle-Dependent Photoelectric Properties in Twisted Bilayer WSe_2_

**DOI:** 10.3390/molecules31101627

**Published:** 2026-05-12

**Authors:** Yunpei Ma, Yuchun Wang, Haiwei Zhang, Jing Yu, Jingang Wang

**Affiliations:** 1 College of Science, Liaoning Petrochemical University, Fushun 113001, China; yunpeima@163.com (Y.M.); yuchun0618@outlook.com (Y.W.); 2 Department of Information Engineering, Fushun Vocational Technology Institute, Fushun 113122, China; 15941309901@163.com

**Keywords:** twisted bilayer WSe_2_, twist angle, moiré superlattice, optical properties, density functional theory (DFT), transition metal dichalcogenides (TMDCs)

## Abstract

The twist angle serves as a geometric tuning parameter in two-dimensional layered materials, enabling modulation of interlayer coupling and band structures without altering the chemical composition. In this work, six commensurate twisted bilayer WSe_2_ configurations with rotation angles of 0°, 9.4°, 13.14°, 21.9°, 27.8°, and 60° were systematically investigated using first-principles density functional theory. Structural optimization, together with calculations of electronic structures, density of states, charge redistribution, effective masses, and optical properties, was performed. The results show that AA (0°) and 2H (60°) stackings exhibit the largest and smallest interlayer separations, respectively, whereas intermediate twist angles yield similar average spacings but distinct local stacking registries. All configurations remain indirect-gap semiconductors, with the valence band maximum located at K and the conduction band minimum near the Q point along the K–Γ path. The band gap increases from 1.450 eV at 0° to 1.579 eV at 27.8°, before decreasing to 1.333 eV at 60°, indicating strong twist-angle modulation of interlayer coupling. Density-of-states analysis shows that the valence-band edge mainly originates from Se-p and W-d hybridized states, whereas the conduction-band edge is dominated by W-d states, with intermediate angles exhibiting enhanced band folding and localization features. Charge-density analyses further reveal notable interfacial charge redistribution, which is most pronounced at 9.4°. Optical responses in the in-plane directions are nearly identical and significantly stronger than those along the out-of-plane direction. Optical absorption mainly occurs in the ultraviolet region, with band-edge features appearing in the near-infrared range. Intermediate twist angles exhibit broader dielectric responses in the visible region and extended long-wavelength tails, indicating enhanced interband transition channels. These results demonstrate that twist-angle engineering enables effective tuning of electronic and optical properties in bilayer WSe_2_, providing theoretical guidance for the design of tunable optoelectronic devices.

## 1. Introduction

Transition metal dichalcogenides (TMDCs) have emerged as central platforms for two-dimensional optoelectronic and quantum regulation studies due to their atomic-scale thickness, strong spin–orbit coupling (SOC) [1,2], and pronounced light–matter interactions. As a representative TMDC material, monolayer WSe_2_ exhibits prominent valley physics and strong excitonic effects. When extended to bilayer or multilayer forms, interlayer van der Waals coupling induces band reconstruction, changes in band-gap character, and enhanced optical anisotropy [3,4]. In this context, the twist angle provides an appealing additional degree of freedom: relative rotation between the two layers generates spatially varying local stacking configurations, forming moiré superlattices that introduce new Brillouin-zone folding patterns in reciprocal space. This process leads to band reconstruction, enhanced density of states, and even the emergence of localized states [5]. Consequently, even without altering chemical composition, purely geometric misalignment can transform an ordinary bilayer into a system with fundamentally modified electronic structures and optical selection rules.

More importantly, twisted WSe_2_ has been systematically realized experimentally. The two limiting stacking configurations, namely 0° and 60°, can be obtained by chemical vapor deposition or epitaxial growth, and the controllable synthesis of 2H bilayers on sapphire substrates has demonstrated good structural stability [6]. In samples with a twist angle of 1.36° close to 0°, eight split moiré intralayer exciton peaks have been observed in the energy range of 1.55–1.73 eV [7]. Subsequently, flat bands were directly resolved by STM/STS in samples with twist angles of 3° and 57.5°, while lattice-reconstruction-induced ultra-flat bands were further observed in near-60° structures with twist angles ranging from 54.1° to 58.4° [8]. For intermediate and large twist angles, commensurate moiré crystals can form at 21.8° and 38.2°, accompanied by dense mini-gaps near the valence-band maximum of the K valley, whereas a 30° twist exhibits a dodecagonal quasicrystalline electronic response [9]. Furthermore, correlated insulating states and zero-resistance pockets have been identified in samples with twist angles of 4–5.1°, and robust superconductivity has been observed in samples with twist angles of 3.5–3.65° [10,11]. These findings indicate that twisted WSe_2_ has evolved from a merely fabricable moiré structural platform into an experimental system of broad interest for band engineering, quasicrystalline physics, and strongly correlated quantum states.

Despite the increasingly clear physical picture of twisted systems, two major challenges remain in practical theoretical investigations [12,13,14,15]. First, small twist angles correspond to extremely large moiré supercells, often requiring prohibitively large computational resources. Second, optical and transport properties are highly sensitive to band-edge details, necessitating a balance among computational feasibility, model commensurability, and physical interpretability [16,17,18]. Therefore, selecting a set of computationally tractable twist angles that represent different stacking symmetries and moiré modulation strengths, and systematically comparing them within a unified computational framework, provides an efficient strategy for constructing the causal relationship between twist angle, interlayer coupling, electronic structure, and optical response [19,20].

Based on the aforementioned experimental advances, twisted bilayer WSe_2_ has been verified across multiple twist-angle regimes, including small-angle moiré excitons, correlated phases at 4–5.1°, ultra-flat bands near 60°, commensurate moiré crystals at large twist angles of 21.8° and 38.2°, and a 30° quasicrystalline structure [9,10,11]. These results indicate that this system has evolved from a merely “fabricable” moiré material into a “tunable and measurable” moiré quantum platform. However, existing studies have still mainly focused on correlated states, flat bands, or local spectroscopic features at only a few specific twist angles. A systematic comparison of the coupled structure–electronic–optical evolution from AA to 2H stacking, spanning intermediate to large twist angles, remains lacking, particularly with regard to a unified physical picture connecting interlayer geometry, local registry, charge redistribution, band folding, and optical response within a single computational framework.

Motivated by this, the present work considers six representative commensurate twisted bilayer WSe_2_ configurations, namely 0° (AA), 9.4°, 13.14°, 21.9°, 27.8°, and 60° (2H). Within a unified first-principles framework including both spin–orbit coupling (SOC) and van der Waals (vdW) corrections [21,22,23], we systematically perform structural optimization, moiré geometry verification, band structure and density-of-states analyses, charge-density-difference and plane-averaged charge-distribution calculations, as well as investigations of effective mass, absorption coefficient, and dielectric function. The novelty of this work lies in three aspects. First, a continuous comparison is established from high-symmetry stackings to intermediate-twist configurations, providing a unified twist-angle-dependent reference. Second, the electronic structure, interfacial charge behavior, and optical response are correlated to reveal how twisting reconstructs the transition channels by modifying interlayer coupling and the distribution of band-edge states. Third, from the perspective of optoelectronic device applications, the functional roles of different twist angles are clarified, thereby providing guidance for material selection and structural design [24,25,26].

Accordingly, this work aims to address several key questions: how the interlayer distance and local stacking evolve with twist angle; why the band gap and the orbital origins of the band edges exhibit systematic variations; how interfacial charge redistribution influences the dielectric and absorption characteristics; and which intermediate twist angles are more suitable as candidate structures for tunable absorbers and optical modulators.

## 2. Results

### 2.1. Unit Cell Structure Analysis

**Figure 1:**
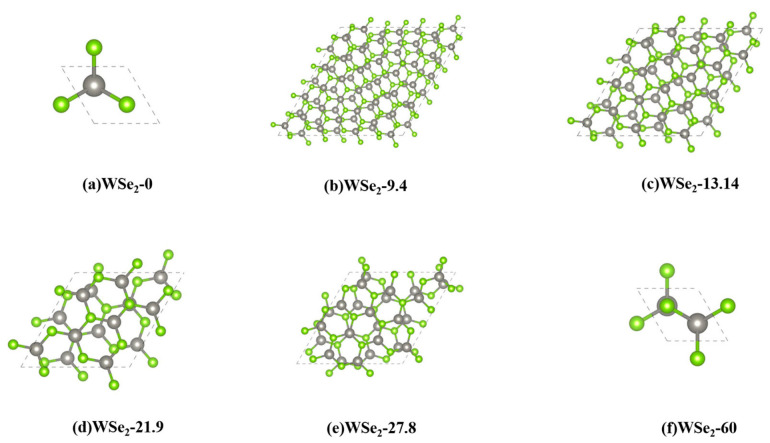
DFT-optimized crystal structures of twisted bilayer WSe_2_ with six representative twist angles: (**a**) 0° (AA), (**b**) 9.4°, (**c**) 13.14°, (**d**) 21.9°, (**e**) 27.8°, and (**f**) 60° (2H). The gray and green spheres represent W and Se atoms, respectively.

Figure 1 and Table 1 present the DFT-optimized crystal structures and corresponding structural parameters of six twisted bilayer WSe_2_ configurations. Considering both computational feasibility and theoretical relevance, six representative twist angles—0°, 9.4°, 13.14°, 21.9°, 27.8° and 60°—were investigated. Previous studies have established that the 60° configuration corresponds to the 2H stacking geometry, in which Se atoms in the upper layer are positioned above W atoms in the lower layer, resulting in relatively reduced interlayer repulsion [27,28,29,30]. In contrast, the 0° configuration corresponds to AA stacking, where Se atoms in the top layer are directly aligned above Se atoms in the bottom layer. Because each atom occupies a finite spatial region, the overlap of electron clouds leads to strong interlayer repulsion. For intermediate twist angles, the relative positions of Se atoms in the upper layer with respect to W atoms in the lower layer vary spatially due to moiré modulation, resulting in mixed local stacking registries [27].

The calculated results show that the interlayer separations are 0.705 nm and 0.650 nm for the 0° and 60° structures, respectively, whereas the remaining twisted configurations exhibit nearly constant interlayer distances around 0.675 nm. These values indicate that the AA-stacked 0° structure possesses the weakest interlayer coupling due to its larger layer separation. In contrast, the 60° configuration exhibits the smallest interlayer spacing, leading to stronger van der Waals attraction, enhanced interlayer orbital overlap, and consequently stronger interlayer coupling compared with the other twist-angle structures.

**Figure 2:**
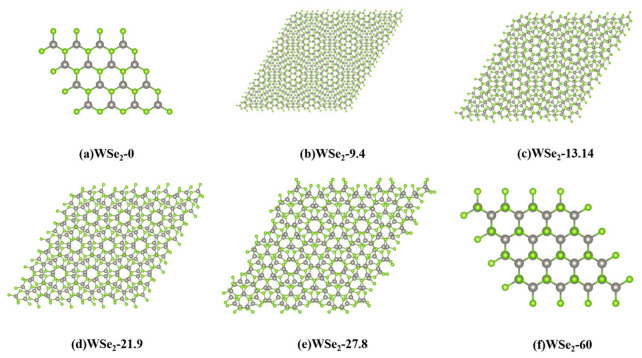
Moiré superlattice structures of twisted bilayer WSe_2_ at different twist angles: (**a**) 0° (AA), (**b**) 9.4°, (**c**) 13.14°, (**d**) 21.9°, (**e**) 27.8°, and (**f**) 60°(2H). The gray and green spheres represent W and Se atoms, respectively.

Figure 2 presents the top-view images of the moiré superlattice structures in twisted bilayer WSe_2_. Owing to the relative rotational misalignment between the two layers, the interlayer atomic registry varies gradually across the in-plane direction, leading to the formation of a periodic structure much larger than the primitive unit cell, commonly referred to as a moiré superlattice. Within the tilted parallelogram-shaped supercell, large-scale repeating patterns emerge, which are characteristic features of moiré states. The calculated moiré lattice constants *a*_moiré_ at twist angles of 0°, 9.4°, 13.14°, 21.9°, and 60° agree well with the geometric predictions given by the equation

(1) $$a_{\mathrm{moire}}\approx \frac{a_{0}}{2\sin(\theta /2)}$$

where *a*_0_ denotes the lattice constant of the primitive cell [28,29,30,31]. This agreement confirms the correctness of the constructed supercells and demonstrates consistency with standard moiré geometric relations.

The structural parameters listed in Table 1 further confirm the validity of the constructed supercells. Taking the primitive lattice constant $a_{0}\approx 3.288$ Å as the reference, and combining it with Equation (1) to estimate the moiré period, the theoretical values for twist angles of 9.4°, 13.14°, and 21.9° are approximately 20.06 Å, 14.37 Å, and 8.65 Å, respectively. These values are in excellent agreement with the optimized lattice constants of 20.003 Å, 14.336 Å, and 8.699 Å listed in the table, indicating that the supercell sizes of these intermediate-twist structures are well consistent with the geometric predictions [28,29]. This agreement also demonstrates that the constructed commensurate moiré supercells can accurately capture the long-period modulation induced by interlayer rotation.

By contrast, 0° and 60° correspond to the two high-symmetry limiting configurations, with lattice constants of 3.288 Å and 3.289 Å, respectively, both of which are essentially equal to the primitive lattice constant. This indicates that the system no longer exhibits long-period moiré modulation in these two cases, but instead reduces to the primitive-cell limits of AA and 2H stacking, respectively. It should be noted that the lattice constant of the 27.8° structure is 11.854 Å, which does not decrease monotonically according to the continuous small-angle geometric relation, but is significantly larger than the directly estimated geometric value. This suggests that the supercell size at this angle is governed primarily by discrete commensurability conditions, reflecting the jump-like behavior between different integer matching solutions [9]. Overall, the lattice constants listed in Table 1 are consistent with both the moiré geometric prediction and the commensurate construction rule, thereby verifying the correctness of the supercell models and indicating that the structural parameters at different twist angles have clear geometric origins and physical significance.

Compared with the 9.4° configuration, the moiré periods at 13.14° and 21.9° become significantly smaller, resulting in correspondingly reduced supercell sizes and fewer atoms in the constructed structures. Within a single moiré supercell, multiple local stacking regions, including AA-WSe_2_, AB-WSe_2_, and BA-type WSe_2_ registries, coexist, which can be energetically regarded as an effective average stacking configuration [26,27,28,29,30,31,32]. Consequently, the interlayer distance converges to an intermediate value, leading to only minor variations among the 9.4°, 13.14°, 21.9°, and 27.8° structures, where the interlayer spacing remains within 0.674–0.677 nm. This behavior is consistent with the interlayer-distance trends discussed previously.

### 2.2. Band Structures and Brillouin Zones

**Figure 3:**
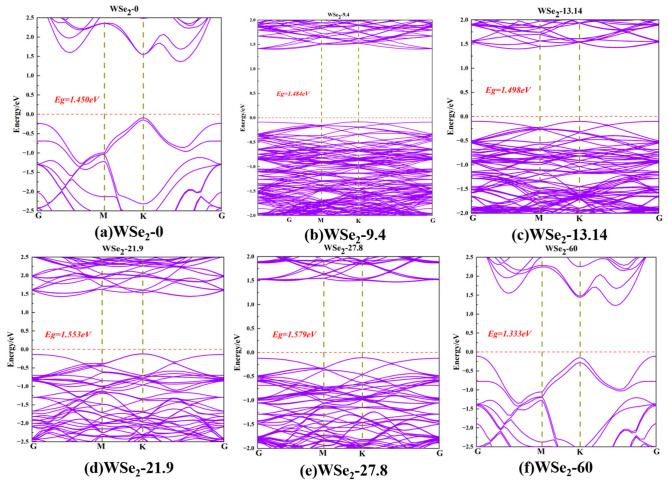
Electronic band structures of twisted bilayer WSe_2_ at different twist angles: (**a**) 0°, (**b**) 9.4°, (**c**) 13.14°, (**d**) 21.9°, (**e**) 27.8° and (**f**) 60°.

All six twisted bilayer WSe_2_ configurations are found to exhibit semiconducting behavior [29,30,31]. As shown in Figure 3a–f, the calculated band gaps for twist angles of 0°, 9.4°, 13.14°, 21.9°, 27.8°, and 60° are 1.450, 1.484, 1.498, 1.553, 1.579, and 1.333 eV, respectively. As the twist angle increases from 0° to 27.8°, the band gap shows a continuous increase, reaching a maximum value of 1.579 eV at 27.8° (Figure 3e). Meanwhile, the presence of the moiré superlattice leads to pronounced band folding and the formation of dense miniband features near the band edges, indicating reduced band dispersion and an increase in carrier effective masses [32]. In contrast, the 60°(2H) stacking exhibits a distinct band-edge reconstruction, and as the energetically most stable configuration, its band gap decreases to 1.333 eV. All six twisted bilayer structures exhibit indirect band gaps, with the conduction-band minimum (CBM) located near the Q point along the K–Γ direction and the valence-band maximum (VBM) located at the K point. Due to spin–orbit coupling, band splitting occurs at the K valley in all configurations. In particular, the splittings at the K point are especially pronounced for the 0° and 60° structures, with splitting energies of ΔE = 120 meV and ΔE = 250 meV respectively. The extent to which SOC affects different twist-angle configurations is not determined solely by the supercell size, but is governed primarily by the twist-induced local symmetry and the strength of interlayer hybridization [31,32,33,34,35]. The K-valley states in WSe_2_ inherently exhibit strong atomic SOC and spin–valley locking [3,4]. Therefore, the highly symmetric 0° and 60° configurations are more capable of preserving well-defined K-valley characteristics, making the corresponding splitting easier to resolve directly. By contrast, for intermediate twist angles, the moiré potential significantly enhances band folding and miniband hybridization, leading to a much denser distribution of bands near the K point. As a result, the SOC-induced splitting is more easily obscured in the spectra and thus appears to be weaker. In other words, this difference arises primarily from the twist-induced modification of interlayer coupling and local symmetry, and is only secondarily modulated by the supercell size and the degree of band folding [33,34,35,36,37,38,39,40,41,42,43,44,45,46].

Within the PBE + SOC framework adopted in this work, the band gaps of the six twisted bilayer WSe_2_ configurations are in the range of 1.333–1.579 eV, all exhibiting an indirect-gap character with the valence-band maximum (VBM) at the K point and the conduction-band minimum (CBM) located near Q. In terms of the overall trend, this result is consistent with the experimental understanding that twisting can continuously modulate the near-Fermi-level band structure of WSe_2_. However, in terms of the absolute values, the difference between the calculated gaps and the true electronic band gaps should be carefully noted. At present, direct experimental measurements of the full CBM–VBM electronic gap in twisted bilayer WSe_2_ remain limited, while most existing studies have focused on valence-side minibands, flat bands, or optical excitons. The more explicit scanning tunneling spectroscopy (STS) results available so far indicate that the electronic gap of untwisted bilayer WSe_2_ is about 2.14 ± 0.05 eV, whereas that of near-60° twisted bilayer WSe_2_ at 54.1° is about 2.04 eV [10]. In comparison, the PBE band gaps obtained in this work are lower by approximately 0.46–0.81 eV.

From the perspective of previous theoretical studies, however, the present values still fall within the reasonable range of comparable GGA/PBE-level results. McCreary et al. reported in their first-principles study of 2H/3R bilayer WSe_2_ that the single-particle band gap of the 2H “A” structure is about 1.402 eV, while that of the 3R structure is 1.393 eV, with a difference of only about 9 meV [25]. This suggests that, within a conventional GGA description, the band-gap differences between different stackings are usually small, whereas the absolute values are generally underestimated [21,22,23,24,25]. In addition, ab initio studies oriented toward device transport have also reported an indirect gap of about 1.36 eV [34] for bilayer WSe_2_, which is of the same order as the 1.333 eV obtained here for the 60° configuration. Therefore, the PBE results presented in this work are credible in terms of the relative twist-angle-dependent trends, but they should not be directly regarded as final values quantitatively equivalent to the experimental electronic gap [27,28,29,31,45].

The underestimation of the band gap by the PBE functional originates from the absence of the derivative discontinuity of the exchange-correlation potential in the Kohn–Sham eigenvalues [21,22,23,24,25,26,27,28,29,30,31,32,33]. As a result, generalized gradient approximation functionals usually exhibit a systematic underestimation of the band gaps of semiconductors and insulators. For two-dimensional vdW semiconductors such as WSe_2_, this issue becomes even more pronounced. On the one hand, quasiparticle self-energy corrections tend to increase the true electronic band gap; on the other hand, strong exciton binding lowers the optical transition energy, such that the PBE gap may sometimes appear closer to the optical peak position than to the true electronic gap [33,34,35,36,37].

Hybrid functionals, such as HSE06, can usually improve the underestimation of the band gap to some extent, but their application to this class of materials still has evident limitations [33,34]. First, HSE06 employs a fixed fraction of short-range exact exchange and a fixed screening length, making it difficult to fully capture the pronounced anisotropy, environment-dependent dielectric screening, and variations in interlayer coupling in two-dimensional layered materials [33,34,35,36,37,38,39]. Therefore, for WSe_2_ systems with different thicknesses, stackings, or twist angles, a single set of parameters may not always be universally applicable. Second, recent methodological studies have shown that range-separated hybrid functionals or nonempirically tuned screened range-separated hybrid functionals, which can properly describe long-range dielectric screening, are generally more suitable than standard HSE06 for band-gap prediction [33,34]. This indicates that fixed-parameter hybrid functionals are not necessarily optimal for vdW materials.

Finally, for the moiré supercells considered in this work, which include SOC, vdW corrections, and up to several hundred atoms, the computational cost would increase dramatically if HSE or even GW calculations were further employed [20,21,22]. This is also an important reason why high-accuracy quasiparticle band-gap results for precisely defined twist angles remain relatively scarce. Therefore, the main value of the present PBE + SOC calculations lies in reliably revealing the relative twist-angle-dependent evolution, including the band-gap variation, migration of band-edge positions, enhanced K-valley splitting, and the tendency toward flatter band-edge states, rather than in providing the final absolute electronic band gaps for direct comparison with experiment [39,40,41,42,43,44,45,46].

**Figure 4:**
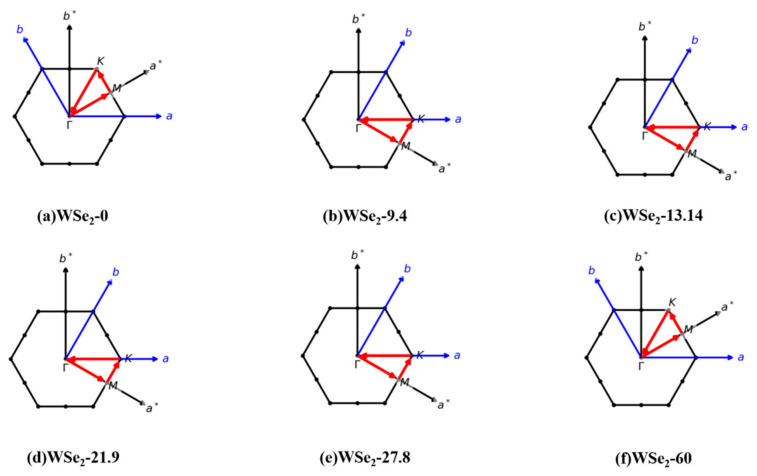
Electronic band structures along the K-point path for twisted bilayer WSe_2_ at different twist angles: (**a**) 0°, (**b**) 9.4°, (**c**) 13.14°, (**d**) 21.9°, (**e**) 27.8°, and (**f**) 60°.The black hexagon represents the first Brillouin zone. The blue arrows indicate the directions of the real-space supercell lattice vectors a and b, while the gray arrows denote the corresponding reciprocal lattice vectors a* and b*. The red polyline marks the high-symmetry path Γ–M–K–Γ used for the band-structure calculations.

Figure 4 illustrates the first Brillouin zones (BZs) of the commensurate moiré supercells and the corresponding high-symmetry K-point paths for twisted bilayer WSe_2_ at twist angles of θ = 0°, 9.4°, 13.14°, 21.9°, 27.8°, and 60°. The black hexagons represent the first BZ of each structure. The blue arrows denote the directions of the real-space supercell lattice vectors a and b, while the gray arrows indicate the corresponding reciprocal lattice vectors a* and b*. The red polyline marks the standard high-symmetry path Γ–M–K–Γ used for band-structure calculations.

Although the geometric orientation of the high-symmetry path in reciprocal space varies with twist angle, each path remains symmetry-equivalent within the Brillouin zone of the corresponding moiré supercell. This ensures the physical consistency and comparability of the calculated band structures across different twist angles and establishes a consistent reciprocal-space framework for analyzing the evolution of electronic structures under moiré modulation.

### 2.3. DOS

In all DOS panels, the dashed line marks the Fermi level E_F_ = 0 eV, around which the total density of states (TDOS) remains nearly zero except for minor broadening tails, confirming that all twisted configurations exhibit semiconducting rather than metallic behavior within the considered energy window. Moreover, the DOS profiles of opposite spin channels are nearly perfectly symmetric, indicating spin degeneracy and the absence of appreciable spin polarization. Therefore, all twisted bilayer WSe_2_ configurations remain in a nonmagnetic or weakly magnetic ground state. The energy difference between the DOS onsets at the valence-band maximum (VBM) and the conduction-band minimum (CBM) defines the band-gap width, which lies in the range of approximately 1.3–1.6 eV for all configurations. This agrees well with the band-structure results discussed previously, where the DOS gap around the Fermi level directly corresponds to the calculated band gaps.

**Figure 5:**
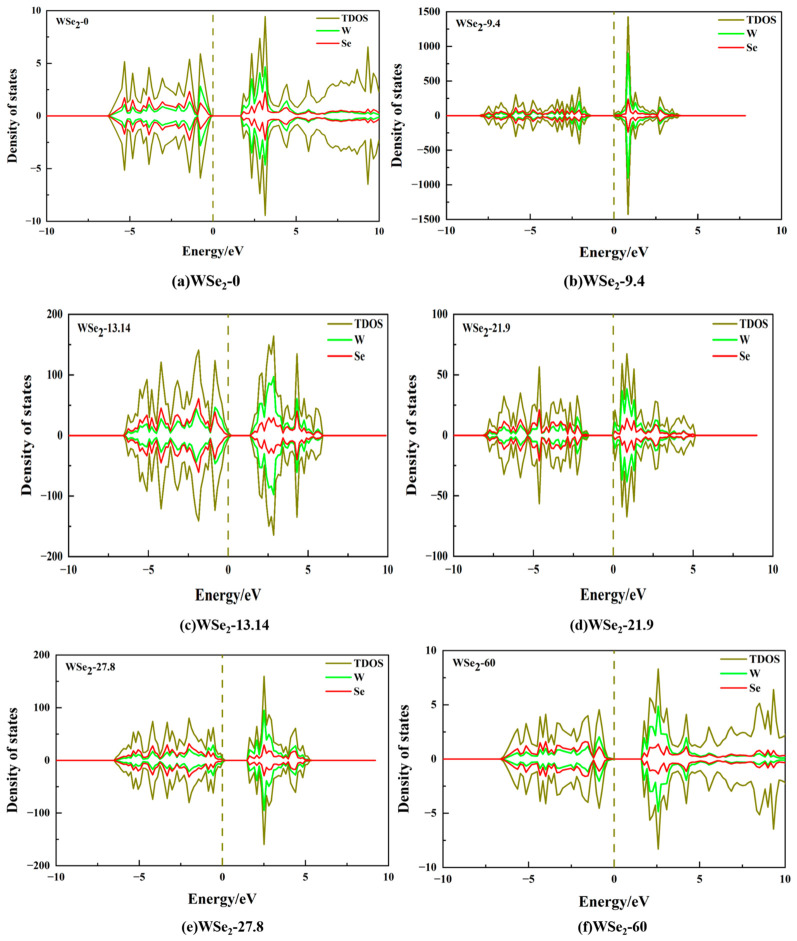
Total density of states (TDOS) and atom-projected density of states (PADOS, W and Se contributions) of twisted bilayer WSe_2_ at different twist angles: (**a**) 0°, (**b**) 9.4°, (**c**) 13.14°, (**d**) 21.9°, (**e**) 27.8°, and (**f**) 60°.

At the atom-projected level (green for W atoms and red for Se atoms), Figure 5a–f show that in the valence-band region, particularly within −3 to 0 eV near the VBM, Se atoms contribute more prominently, while W atoms also participate through hybridized states but with relatively smaller weight. This observation is consistent with the typical electronic structure of WSe_2_, where the VBM mainly originates from hybridization between Se p orbitals—especially in-plane p states—and W d orbitals, with Se contributions usually being more pronounced. In contrast, in the conduction-band region, several prominent peaks appear roughly 1–4 eV above the CBM, where the W contribution closely follows the TDOS, whereas the Se contribution is comparatively weaker. This behavior indicates that the CBM is primarily dominated by W d states, with Se states contributing through secondary hybridization.

A comparison of DOS peak shapes and positions across different twist angles reveals a clear twist-angle dependence. Intermediate twist angles (9.4°, 13.14°, 21.9°, and 27.8°) exhibit sharper DOS peaks, whereas high-symmetry stacking configurations display broader and smoother DOS distributions. As shown in Figure 5a,f, corresponding to the 0° (AA) and 60° (2H) structures, respectively, both TDOS and PADOS features are relatively broadened, indicating stronger band dispersion and more delocalized electronic states, with no extremely sharp singular peaks near the band edges. This behavior is consistent with more uniform interlayer coupling and broader band dispersion in high-symmetry stacking configurations. By contrast, intermediate twist angles exhibit pronounced sharp peaks and structured peak groups on the conduction-band side, demonstrating that moiré-induced band reconstruction remains significant. Differences in peak positions further indicate that twist-angle variation modifies interlayer hybridization strength and moiré Brillouin-zone folding patterns. In particular, the 9.4° configuration exhibits the sharpest DOS peaks, suggesting strong band flattening in certain energy ranges, resulting in DOS values approaching ~1500 at specific energies, whereas the other intermediate angles show comparatively less pronounced peak intensities. From a physical perspective, sharp peaks in the density of states (DOS) usually correspond to flatter bands, stronger state localization, or van Hove singularities, whereas a smoother DOS generally implies stronger band dispersion and more delocalized electronic states [47,48,49,50,51]. For twisted bilayer WSe_2_, the sharper DOS peaks observed at intermediate twist angles indicate that the moiré potential significantly flattens part of the band structure. This effect is favorable for enhancing the density of band-edge states, increasing the probability of optical transitions, and suggesting a higher sensitivity of the system to external doping or gate bias. For electrical conductivity, however, a higher DOS peak does not necessarily imply stronger conduction, because flat bands are often accompanied by lower group velocities and larger effective masses. Therefore, the actual transport properties still depend on the combined effects of the DOS, carrier velocity, and relaxation time.

### 2.4. Charge-Density Difference and Planar-Averaged Charge Density

**Figure 6:**
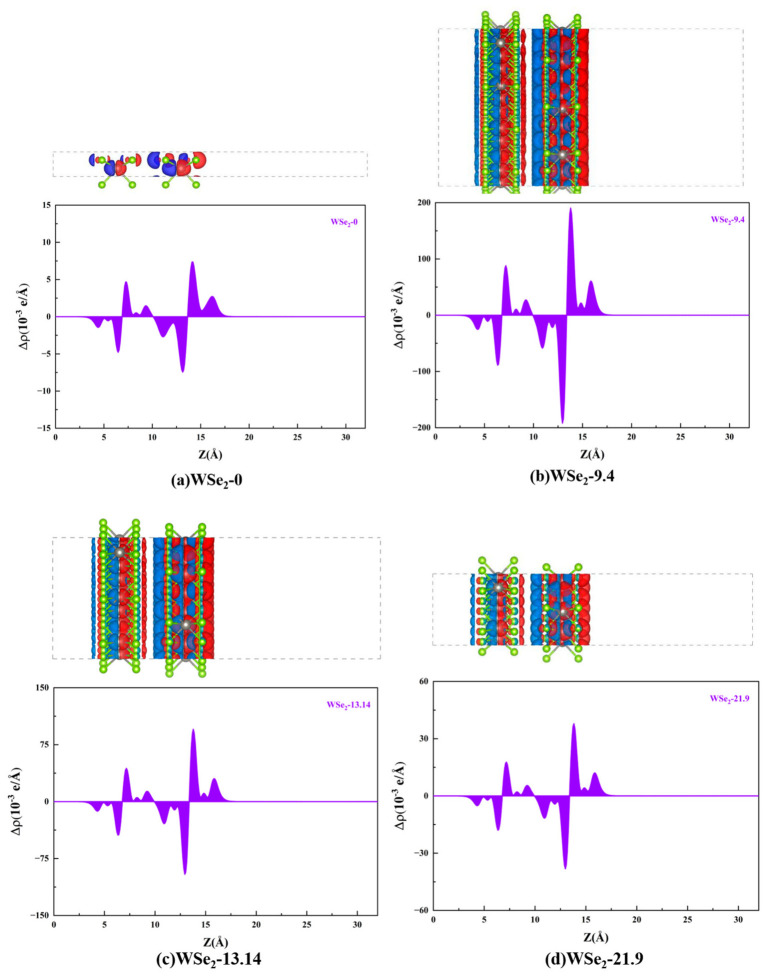

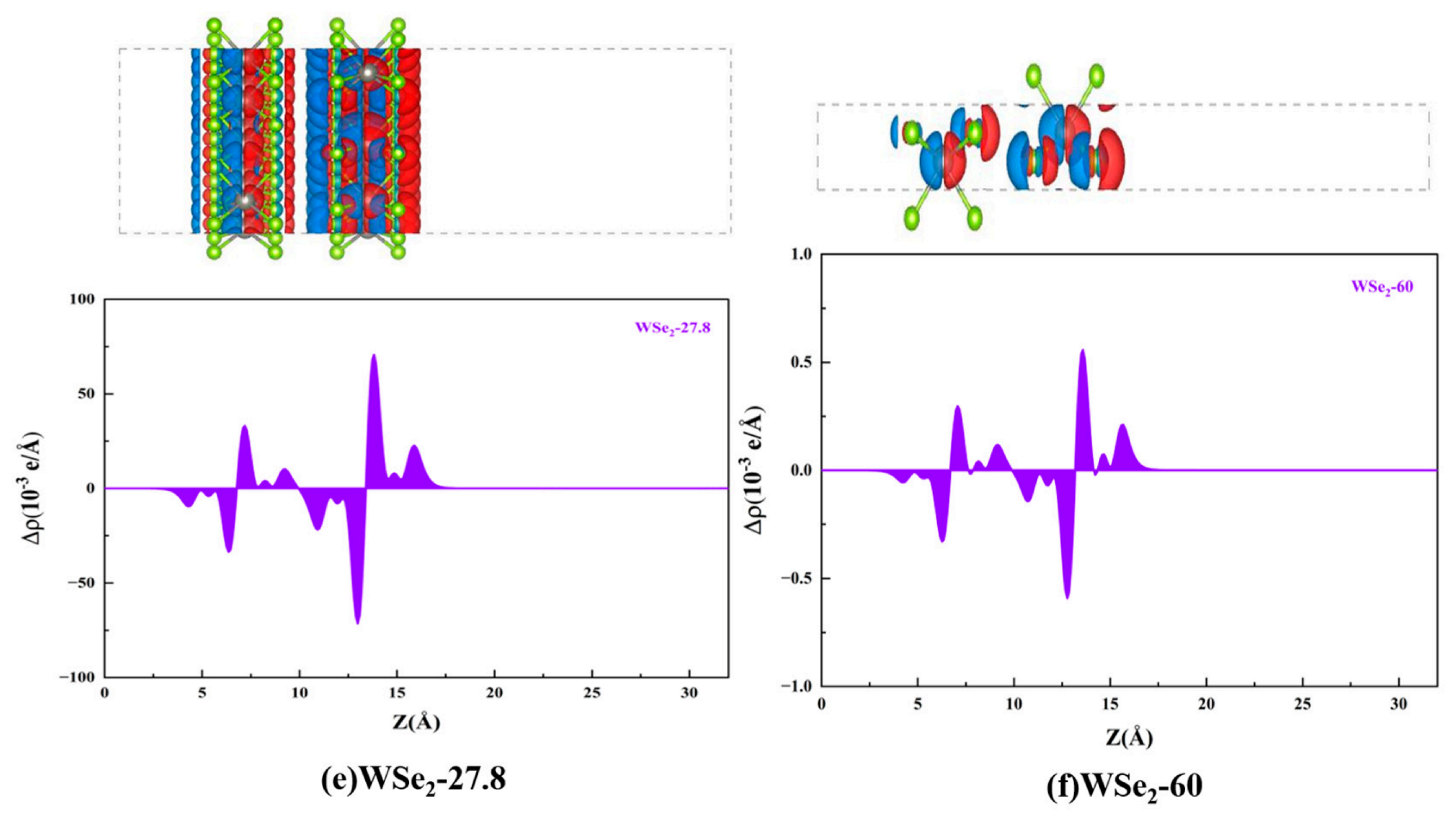
Charge-density difference distributions and corresponding planar-averaged charge densities of twisted bilayer WSe_2_ at different twist angles: (**a**) 0°, (**b**) 9.4°, (**c**) 13.14°, (**d**) 21.9°, (**e**) 27.8°, and (**f**) 60°. The gray and green spheres represent W and Se atoms, respectively. The red and blue isosurfaces denote electron accumulation and electron depletion, respectively. The purple filled curve represents the planar-averaged charge-density difference, Δρ(z), along the z direction.

Figure 6a–f present the charge-density difference distributions and the corresponding planar-averaged charge-density profiles along the out-of-plane [001] direction for twisted bilayer WSe_2_ at different twist angles. These results allow direct visualization of electron accumulation and depletion associated with interlayer charge transfer, where red regions represent electron accumulation and blue regions indicate electron depletion (hole accumulation). The isosurface value is set to 0.0025 for the 0° and 60° configurations, whereas a value of 0.03 is used for the remaining twist angles. For the 0° configuration (Figure 6a, AA stacking), no strongly localized interfacial coupling regions are formed, resulting in relatively weak overall charge redistribution. In contrast, the 9.4° configuration (Figure 6b) exhibits significantly stronger charge redistribution, indicating that twisting enhances interfacial charge rearrangement and strengthens local coupling modulation.

The planar-averaged charge-density difference Δρ(z) shown in Figure 6b further clarifies the spatial redistribution of charge along the out-of-plane direction. In the region around Z ≈ 5–8 Å, positive and negative peaks correspond to charge redistribution within the lower-layer Se–W–Se sandwich structure. In the interfacial region around Z ≈ 8–12 Å, electron depletion (blue regions) is observed near the upper-layer interface, while electron accumulation (red regions) occurs near the lower-layer interface, indicating net electron transfer from the upper to the lower layer, with stronger electron loss on the upper side. At the interface, the amount of electron depletion exceeds that of accumulation, consistent with the features observed in the charge-density difference isosurfaces. In the region around Z ≈ 12–16 Å, more pronounced peak pairs appear, demonstrating that the upper Se–W–Se layer constitutes a primary region of charge redistribution. This behavior further indicates that twist-induced moiré superlattices, together with structural relaxation producing local AB/BA stacking domains and domain boundaries, enhance interlayer orbital overlap and interfacial polarization, leading to stronger spatial charge redistribution and the formation of significant interfacial dipole distributions.

Except for the 60° configuration shown in Figure 6f, all structures exhibit a clear imbalance at the interface, where the amount of electron depletion exceeds that of electron accumulation. Specifically, for the 0° configuration, electron accumulation of 1.47 × 10^−3^ e occurs at (Z = 9.36) Å, while electron depletion of 2.71 × 10^−3^ e appears at (Z = 11.10) Å. For the 9.4° structure, electron accumulation of 27.26 × 10^−3^ e is observed at (Z = 9.23) Å, whereas electron depletion of 58.52 × 10^−3^ e occurs at (Z = 10.91) Å. Similarly, at 13.14°, electron accumulation of 13.57 × 10^−3^ e and depletion of 29.16 × 10^−3^ e appear at (Z = 9.21) Å and (Z = 10.89) Å, respectively. For the 21.9° configuration, the corresponding values are 5.50 × 10^−3^ e at (Z = 9.24) Å and 11.70 × 10^−3^ e at (Z = 10.80) Å, while at 27.8°, electron accumulation of 10.24 × 10^−3^ e and depletion of 21.89 × 10^−3^ e occur at (Z = 9.24) Å and (Z = 10.93) Å, respectively.

Combined with the dielectric-function results shown in Section 2.7, all twisted configurations exhibit pronounced charge accumulation–depletion pairs near the interface, indicating that interlayer coupling induces charge redistribution along the out-of-plane direction and generates interfacial dipoles. Among them, the 9.4° configuration shows the largest peak amplitude, followed by 13.14°, while the effect is weakest at 0°. Consistently, the dielectric spectra in Section 2.7 show that intermediate twist angles, particularly 9.4° and 13.14°, exhibit significantly enhanced in-plane ε_2_ responses in the visible region, together with broader peak features and extended long-wavelength tails. This behavior indicates that interfacial charge redistribution strengthens interlayer hybridization and increases the number of allowed interband transition channels. Correspondingly, ε_1_ displays stronger dispersion in adjacent spectral regions and an elevated low-energy dielectric background, reflecting enhanced polarization and screening capabilities. In contrast, for the 0° and 60° configurations, interfacial charge redistribution is much weaker, leading to overall reduced ε_1_ and ε_2_ responses.

### 2.5. Carrier Effective Masses

**Figure 7:**
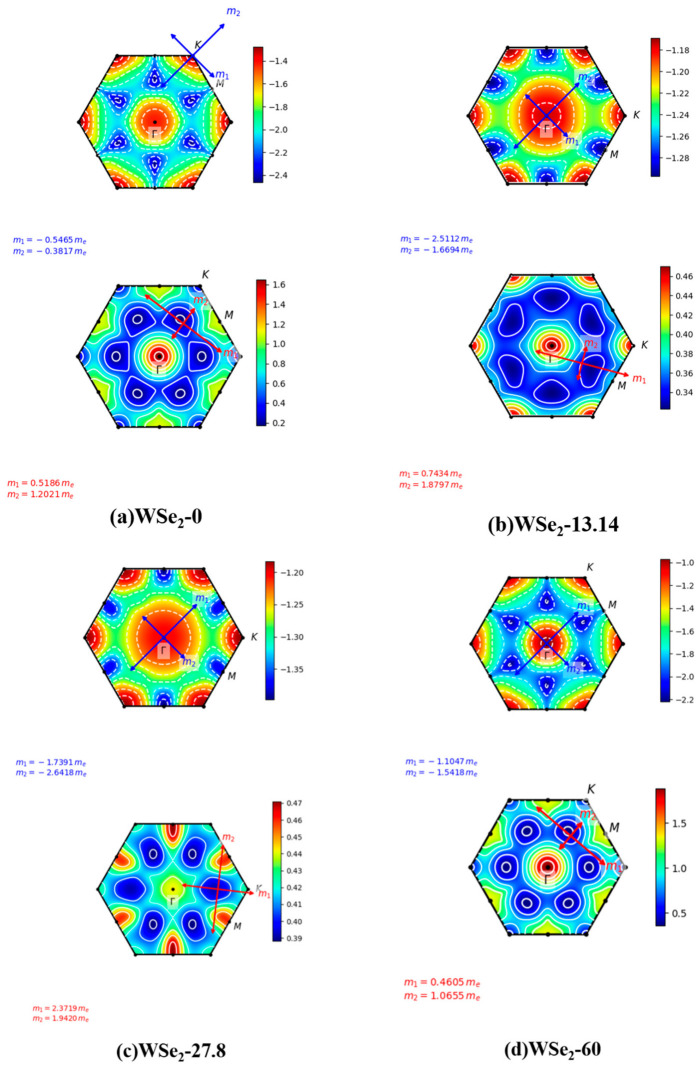
Carrier effective masses of twisted bilayer WSe_2_ at different twist angles: (**a**) 0°, (**b**) 13.14°, (**c**) 27.8°, and (**d**) 60°.

Figure 7a–d display the calculated effective masses of holes and electrons in twisted bilayer WSe_2_, where blue and red symbols represent hole and electron effective masses, respectively. For holes, at a twist angle of 0°, the effective masses are m_1_ = −0.5465 m_e_ and m_2_ = −0.3817 m_e_. Since |m_1_| > |m_2_|, the valence band is flatter along the m_1_ principal direction, leading to heavier holes, while along the m_2_ direction the band dispersion is steeper and holes are relatively lighter. The ratio |m_1_/m_2_| ≈ 1.43 indicates moderate anisotropy. At 13.14°, the effective masses increase to m_1_ = −2.5112 m_e_ and m_2_ = −1.6694 m_e_, indicating that the valence band becomes significantly flatter along both principal directions compared with the 0° case, consistent with the band features shown in Figure 3c. The anisotropy remains moderate, with |m_1_/m_2_| ≈ 1.50. At 27.8°, the hole masses remain large, with m_1_ = −1.7391 m_e_ and m_2_ = −2.6418 m_e_, and the anisotropy is still evident, |m_1_/m_2_| ≈ 1.52. For the 60° configuration, the hole masses decrease to m_1_ = −1.1047 m_e_ and m_2_ = −1.5418 m_e_, becoming lighter than those at intermediate twist angles but still heavier than those at 0°, while the anisotropy remains moderate, |m_1_/m_2_| ≈ 1.40.

For electrons, at a twist angle of 0°, the effective masses are m_1_ = 0.5186 m_e_ and m_2_ = 1.2021 m_e_, with m_2_ > m_1_, indicating that the conduction band is steeper along the m_1_ principal direction, resulting in lighter electrons compared with the m_2_ direction. The ratio m_1_/m_2_ ≈ 2.32 reflects strong transport anisotropy. At 13.14°, both effective masses increase to m_1_ = 0.7434 m_e_ and m_2_ = 1.8797 m_e_, indicating heavier electrons along both principal directions relative to the 0° configuration, particularly along the m_2_ axis. The anisotropy remains strong, with m_1_/m_2_≈2.53. At 27.8°, the effective masses further increase to m_1_ = 2.3710 m_e_ and m_2_ = 1.9420 m_e_, indicating significantly flattened conduction bands, consistent with the band dispersion shown in Figure 3e. However, the anisotropy becomes weaker compared with the 0° and 13.14° cases, with m_1_/m_2_ ≈ 1.22. For the 60° configuration, the effective masses decrease to m_1_ = 0.4605 m_e_ and m_2_ = 1.0655 m_e_, values comparable to those at 0°, and the anisotropy correspondingly returns to a similar level, with m_1_/m_2_ ≈ 2.31.

**Figure 8:**
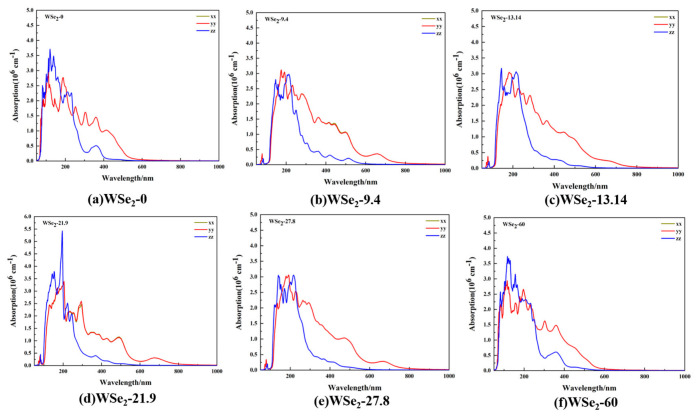
Optical absorption coefficients of twisted bilayer WSe_2_ along the XX, YY, and ZZ directions at different twist angles: (**a**) 0°, (**b**) 9.4°, (**c**) 13.14°, (**d**) 21.9°, (**e**) 27.8°, and (**f**) 60°.

The effective mass directly reflects carrier response capability, since smaller |m_1_| corresponds to higher carrier mobility and easier acceleration under external perturbations. Holes are lightest at 0°, representing a light-carrier regime, with the 60° configuration showing comparable behavior. Similarly, electrons at both 0° and 60° also remain relatively light and exhibit strong anisotropic transport characteristics. Intermediate twist angles display broader visible-light optical responses and longer long-wavelength tails, whereas the responses at 0° and 60° appear comparatively more confined. The relatively small effective masses at 0° correspond to steeper band-edge dispersions, preventing excessive accumulation of states near the band edges in the DOS. Consistently, the absorption spectrum in Figure 8a shows more compact peak features in the visible region and weaker long-wavelength tails. In contrast, the increased effective masses at 13.14° indicate flatter band edges and enhanced carrier localization, corresponding to the broader visible-light response and more pronounced long-wavelength tails observed in Figure 8c. Likewise, the significantly heavier conduction-band electrons at 27.8° correlate with the broadened visible absorption band and extended long-wavelength tail shown in Figure 8e.

### 2.6. Optical Absorption Coefficients

Figure 8a–f show the calculated optical absorption coefficients of twisted bilayer WSe_2_ in the wavelength range of 0–1000 nm for twist angles of 0°, 9.4°, 13.14°, 21.9°, 27.8°, and 60°. For all configurations, the absorption spectra along the in-plane XX and YY directions nearly overlap, indicating that the in-plane optical response remains close to hexagonal symmetry. Strong optical absorption is mainly concentrated in the ultraviolet region (100–300 nm), while the absorption coefficient gradually decreases in the visible range (300–600 nm). The absorption edge appears approximately in the range of 700–930 nm, consistent with the band-gap values obtained from the band structures shown in Figure 3a–f.

For the 0° configuration (AA stacking), pronounced absorption peaks appear near 110 nm and 190 nm, with absorption coefficients of approximately 3.10 × 10^6^ cm^−1^ and 2.70 × 10^6^ cm^−1,^ respectively. The 60° configuration (2H stacking) exhibits a similar spectral profile, with strong peaks near 115 nm and 195 nm, reaching absorption coefficients of about 2.93 × 10^6^ cm^−1^ and 2.65 × 10^6^ cm^−1^. For the 9.4° structure, a strong absorption peak appears within 170–190 nm, with an intensity of approximately 3.04 × 10^6^ cm^−1^. At 13.14°, the maximum absorption peak occurs near 180 nm with a magnitude of about 3.02 × 10^6^ cm^−1^. In the 21.9° configuration, pronounced peaks are observed near 200 nm and 290 nm, reaching approximately 3.38 × 10^6^ cm^−1^ and 2.59 × 10^6^ cm^−1^, respectively. For the 27.8° configuration, the strongest peak appears near 190 nm with an intensity of about 3.05 × 10^6^ cm^−1^. Along the out-of-plane ZZ direction, both 0° (AA stacking) and 60° (2H stacking) exhibit their strongest absorption peaks near 120 nm, with secondary peaks around 230 nm, after which the absorption coefficient gradually decreases with increasing wavelength. For intermediate twist angles of 9.4°, 13.14°, and 27.8°, prominent peaks appear near 145 nm and 215 nm. In contrast, the 21.9° configuration shows a particularly strong peak around 195 nm, with higher intensity than those of other twist angles. These variations indicate that twisting modifies interlayer hybridization and band folding, thereby influencing band-gap characteristics and the positions and intensities of absorption peaks.

### 2.7. Real Part of the Dielectric Function and Imaginary Part of the Dielectric Function

**Figure 9:**
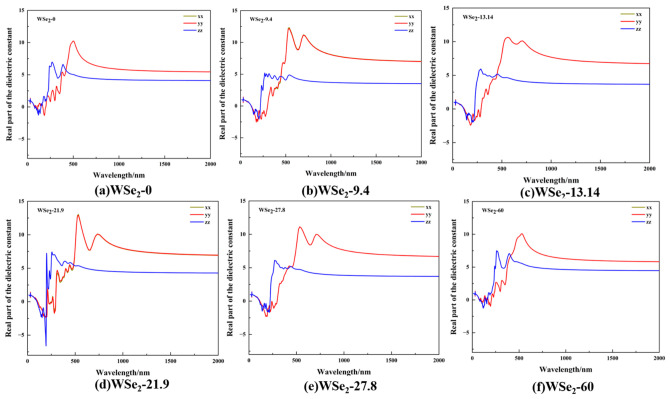
Real part of the dielectric function of twisted bilayer WSe_2_ along the XX, YY, and ZZ directions at different twist angles: (**a**) 0°, (**b**) 9.4°, (**c**) 13.14°, (**d**) 21.9°, (**e**) 27.8°, and (**f**) 60°.

The real part of the dielectric function ε_1_ shown in Figure 9a–f along the XX, YY, and ZZ directions, characterizes the dispersion behavior of the system and determines the general trend of the refractive index. From the strong overlap of the curves, the dielectric responses along the XX and YY directions are nearly identical, indicating that the in-plane optical response is approximately isotropic. The most pronounced ε_1_ features appear in the visible to near-ultraviolet region (200–700 nm), where the in-plane components (XX and YY) exhibit broader responses than the out-of-plane component (ZZ). This behavior is consistent with the orbital characteristics and optical selection rules in layered TMDC materials, where in-plane polarization more effectively excites dominant interband transitions, while out-of-plane dipole matrix elements are typically smaller and their spectral responses are more concentrated in the deep ultraviolet region.

Within the wavelength range of 300–700 nm, ε_1_ exhibits clear peak features along the XX and YY directions, although both peak intensity and spectral shape vary significantly with twist angle. For the 0° and 60° configurations, the XX and YY peaks are relatively moderate, with intensities around 10, and the curves return to smoother dispersion plateaus. This behavior is consistent with the expectation that high-symmetry stacking configurations possess more regular interlayer coupling and fewer additional optical transition channels. In contrast, the 9.4° configuration exhibits the highest peaks along the XX and YY directions, reaching values of approximately 12–13, with broader spectral features, indicating enhanced interband transition strength and a wider distribution of transition energies, consistent with the DOS features observed in Figure 5b. Similarly, the 21.9° configuration also shows pronounced in-plane peaks approaching values of ~13, accompanied by sharper structures, suggesting that moiré-induced miniband effects may further concentrate transition channels at this angle. The 13.14° and 27.8° configurations exhibit intermediate peak intensities, typically around 11. In the longer-wavelength range of 1500–2000 nm, the ε_1_ values for intermediate twist angles (9.4°, 13.14°, 21.9°, and 27.8°) are generally higher than those of the 0° and 60° configurations. This indicates stronger low-energy polarization and effective screening in intermediate-angle structures, which, at the device level, correspond to higher low-frequency dielectric backgrounds and larger refractive-index baselines. Such characteristics provide useful guidance for the design of twist-engineered optoelectronic devices, including tunable absorbers and optical modulators.

**Figure 10:**
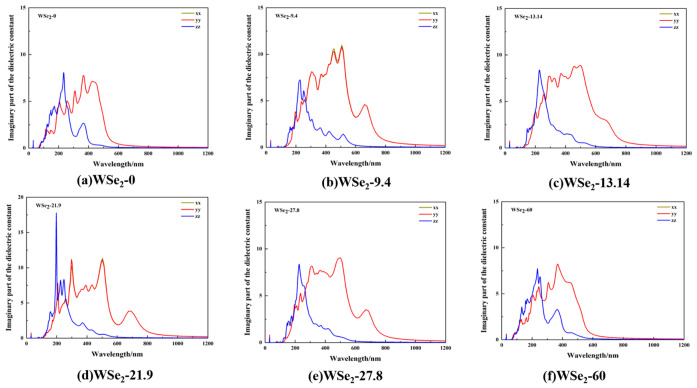
Imaginary part of the dielectric function of twisted bilayer WSe_2_ along the XX, YY, and ZZ directions at different twist angles: (**a**) 0°, (**b**) 9.4°, (**c**) 13.14°, (**d**) 21.9°, (**e**) 27.8°, and (**f**) 60°.

For all considered twist angles, the imaginary part of the dielectric function ε_2_ shown in Figure 10a–f, directly reflects the strength and energy distribution of interband optical transitions. Twist modulation primarily affects peak intensity, spectral broadening, and the extent of long-wavelength tails. A common feature among all configurations is strong two-dimensional anisotropy, with the in-plane components (XX and YY) exhibiting significantly stronger responses than the out-of-plane component (ZZ). Consistent with the behavior of the real part of the dielectric function in Figure 9a–f, the XX and YY components nearly overlap, indicating an approximately isotropic in-plane optical response. In contrast, strong peaks along the ZZ direction are mainly concentrated in the ultraviolet region (150–300 nm) and rapidly decay to nearly zero upon entering the visible range. This behavior reflects the layered nature of the system, where out-of-plane polarization is less effective in exciting low-energy optical transitions and primarily contributes to higher-energy transitions.

For the 0° and 60° configurations, the ε_2_ spectra along the XX and YY directions are comparatively smoother than those of twisted configurations, with visible-region peaks more concentrated and gradually approaching zero near 900 nm. In the 9.4° configuration, broader and stronger peak groups appear in the 350–600 nm range along the XX and YY directions, with peak values reaching approximately 12 in Figure 10b. Moreover, pronounced spectral tails persist in the 700–900 nm region, demonstrating that twist-induced band folding not only enhances allowed transition channels but also distributes them over a wider energy range. For the 13.14° and 27.8° configurations, noticeable spectral broadening remains along the in-plane directions, although peak intensities decrease to around 8, while long-wavelength tails remain more pronounced than those of the 0° and 60° cases. At 21.9°, strong in-plane peaks with sharper and more structured features are observed, consistent with the sharp dispersion behavior in Figure 9d. Along the ZZ direction, a pronounced deep-ultraviolet peak around 200 nm suggests stronger interlayer-coupling-induced transitions at this twist angle.

**Figure A2:**
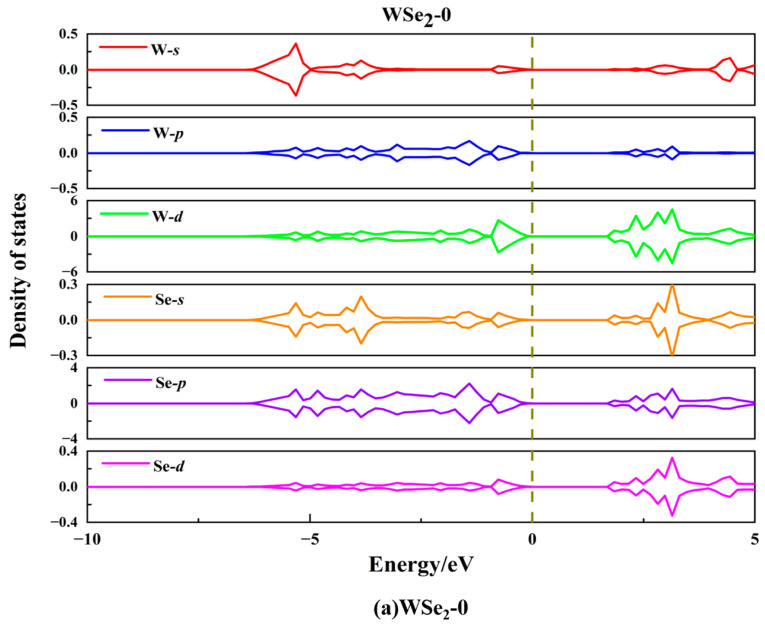

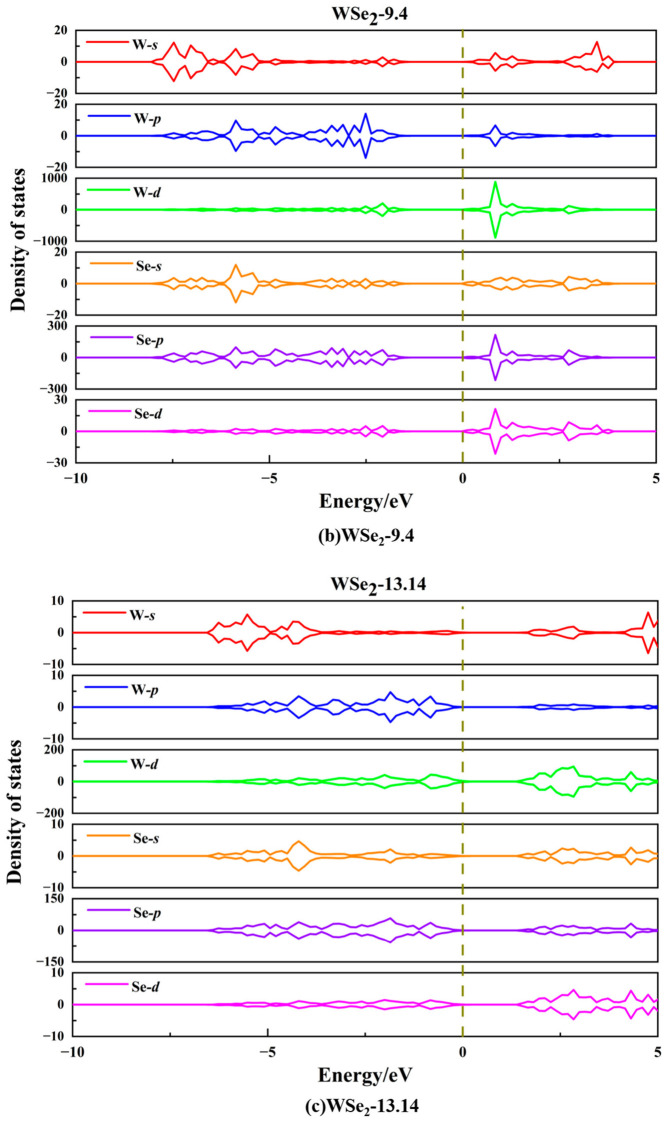

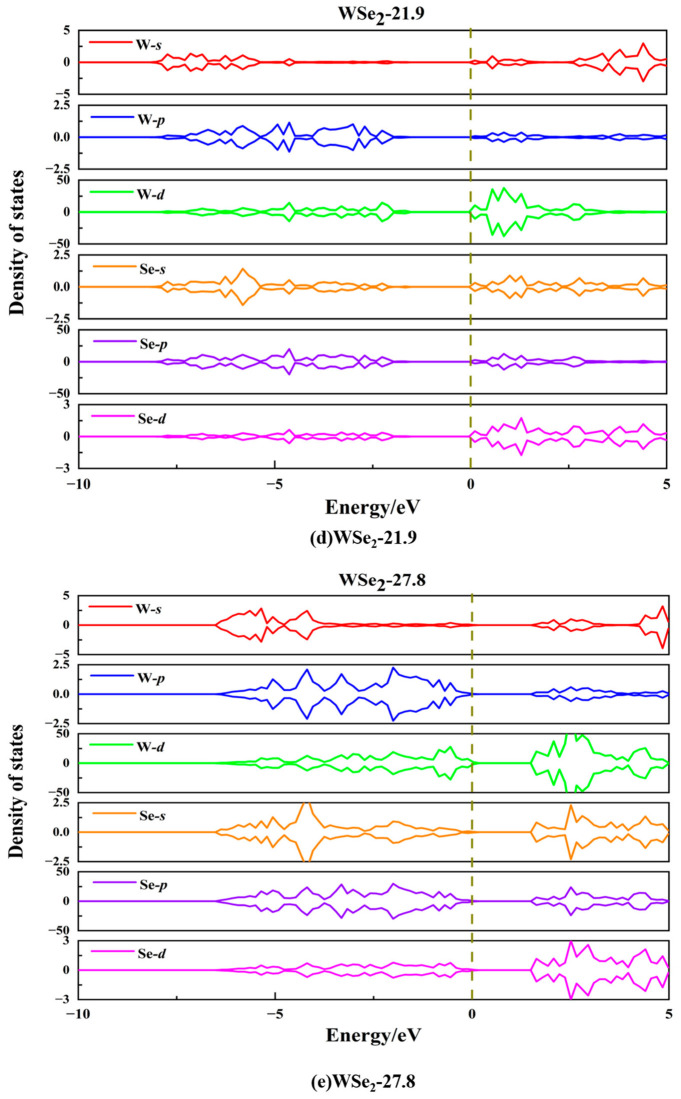

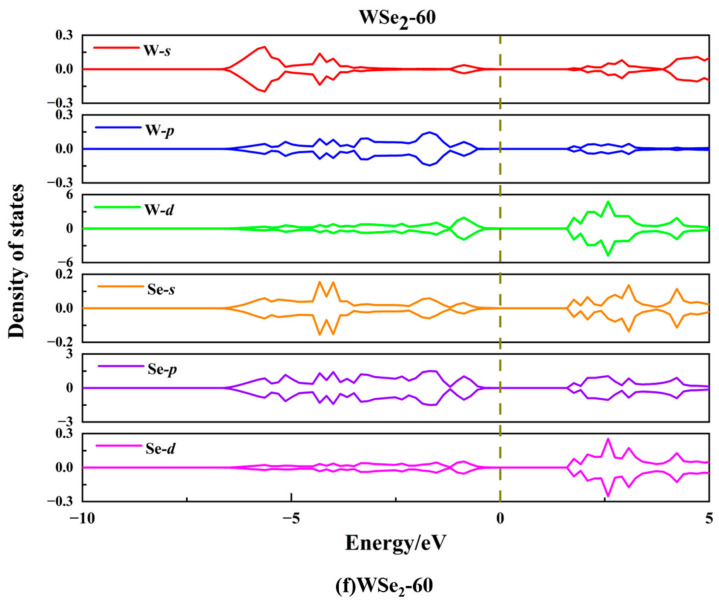
Orbital-projected density of states (ODOS) of W and Se atoms in twisted bilayer WSe_2_ at different twist angles: (**a**) 0°, (**b**) 9.4°, (**c**) 13.14°, (**d**) 21.9°, (**e**) 27.8°, and (**f**) 60°.

By combining the DOS results in Figure 5 and Figure A2 with the ε_2_ spectra in Figure 10, it is evident that the dominant optical transitions originate from interband transitions between Se-derived valence states (approximately corresponding to Se-4p orbitals) and W-derived conduction states (associated with W-5d orbitals). At a twist angle of 9.4°, sharp W-dominated DOS peaks appear near the conduction-band edge, indicating that moiré-induced localization significantly increases the density of states participating in optical transitions within a narrow energy window. Together with enhanced Se-4p/W-5d hybridized states near the valence-band edge, this leads to strong and broadened ε_2_ peaks in the visible region (approximately 400–550 nm, corresponding to 3.10–2.25 eV), accompanied by pronounced shoulder features and extended absorption tails at longer wavelengths. In contrast, DOS peaks at 0° and 60° are more dispersed, resulting in weaker and narrower ε_2_ peaks in the visible range, while the 13.14° and 27.8° cases exhibit intermediate behavior. Notably, the 21.9° configuration exhibits a sharp and intense ε_2_ peak in the deep-ultraviolet region (approximately 200–250 nm, corresponding to 6.2–5.0 eV), indicating that interlayer-coupling-related transitions from deeper Se-4p valence states to higher-energy W-5d conduction states contribute more prominently to the optical response.

## 3. Materials and Methods

The first-principles calculations in this work were performed using the Vienna Ab initio Simulation Package (VASP 6.3.2) within the framework of density functional theory (DFT). The exchange-correlation energy was described by the generalized gradient approximation (GGA) in the Perdew–Burke–Ernzerhof (PBE) form, and the interaction between valence electrons and ionic cores was treated using the projector augmented-wave (PAW) method [52,53,54,55].

To ensure good convergence, the plane-wave cutoff energy was set to 450 eV. A vacuum layer of more than 20 Å was introduced along the *c* direction to eliminate spurious interlayer interactions, and the DFT-D3 method was employed to accurately account for the van der Waals (vdW) interaction [56,57]. Structural relaxations were carried out using the conjugate-gradient algorithm. To balance computational accuracy, efficiency, and parallel performance, Monkhorst–Pack *k*-point meshes of 6×6×1, 3×3×1, and 4×4×1 were adopted for the 0°/60°, 9.4°/13.14°, and 21.9°/27.8° configurations, respectively [58,59]. The k-mesh convergence tests are provided in Appendix A. For all structures, both the lattice parameters and atomic positions were fully relaxed. The convergence criteria for the total energy and the residual forces were set to $10^{-6}$ eV and 0.02 eV/Å, respectively. In addition, spin–orbit coupling (SOC) was included in both the self-consistent charge density calculations and the band-structure calculations [60].

## 4. Conclusions

This work establishes a systematic structure–electronics–optics correlation for six commensurate twisted bilayer WSe_2_ configurations. The central conclusion is that the strength of interlayer coupling is jointly determined by stacking configuration and twist angle, while the resulting physical responses are primarily manifested through band-edge reconstruction, band folding, and redistribution of interband transition channels. From a structural perspective, the 0°(AA) configuration exhibits the largest interlayer separation (0.705 nm), corresponding to the weakest interlayer coupling, whereas the 60° (2H) stacking shows the smallest interlayer distance (0.650 nm), indicating stronger interlayer orbital overlap and enhanced van der Waals cohesion. In contrast, intermediate twist angles (9.4°, 13.14°, 21.9°, and 27.8°) exhibit interlayer separations converging within approximately 0.674–0.677 nm. Although the average geometric spacing varies only slightly in these cases, variations in local stacking registry and moiré superlattice formation still significantly influence the distribution of electronic states.

At the electronic-structure level, all configurations remain indirect-gap semiconductors, with the valence-band maximum consistently located at the K point and the conduction-band minimum situated near the Q point along the K–Γ path. The band gap increases continuously from 0° to 27.8°, reaching a maximum value of 1.579 eV, whereas at 60° the stronger interlayer coupling reduces the gap to 1.333 eV. Meanwhile, moiré-induced band folding becomes particularly pronounced at intermediate twist angles, producing dense miniband structures near the band edges and correspondingly sharper DOS peaks, reflecting reduced band dispersion and enhanced density of states. Orbital-resolved analysis further shows that the valence-band maximum mainly originates from hybridized Se-p and W-d states, while the conduction-band minimum is predominantly W-d in character. Therefore, the essential effect of twist modulation can be understood as changes in interlayer hybridization strength and folding patterns, which modify the relative energy positions and curvature of W-d-dominated conduction-band edges and Se-p-related valence-band edges.

Interfacial charge behavior further supports the above physical picture. Charge-density difference analysis shows that twisting enhances interfacial charge redistribution, with the 9.4° configuration exhibiting the strongest charge accumulation and depletion peaks at the interface, indicating more pronounced local coupling and polarization induced by moiré domains and domain boundaries. Transport-related effective-mass calculations further reveal that holes are lightest at 0°, while they become significantly heavier at 13.14° and 27.8°. For electrons, relatively small effective masses and strong anisotropy are observed at 0° and 60°, whereas at 27.8° electrons become markedly heavier and the anisotropy is reduced, demonstrating that twisting effectively modulates band-edge curvature and thus carrier response capability.

From an optical perspective, absorption mainly occurs in the ultraviolet region, and the nearly overlapping XX and YY components indicate an approximately isotropic in-plane response. The dielectric function exhibits pronounced two-dimensional anisotropy, with in-plane components significantly stronger than the out-of-plane component. In particular, the imaginary part of the dielectric function, ε_2_ shows broader visible-region peak groups (approximately 350–600 nm) and extended long-wavelength tails (700–900 nm) at intermediate twist angles, especially around 9.4°, whereas the spectral responses at 0° and 60° are smoother and more concentrated. These results indicate that the twist angle enhances optical responses by increasing the number of allowed transition channels and broadening their energy distribution.

Overall, this study establishes a practical twist-angle selection strategy: intermediate twist angles (such as 9.4° and 21.9°) are advantageous when broader and stronger visible-region optical transitions and dielectric responses are desired, whereas the 60° (2H) configuration serves as a limiting case when stronger interlayer coupling and distinct band-edge reconstruction are of interest. The present work provides a foundation for future studies incorporating excitonic effects, external-field tuning, or device-level modeling, such as in the design of optical modulators and tunable absorbers.

## Figures and Tables

**Table 1:** Twist angles, number of atoms, lattice constants, and interlayer distances of the six twisted bilayer WSe_2_ structures.

θ/(°)	Number of Atoms *N_a_*	Lattice Constant a/Å	d/nm
0°	6	3.288	0.705
9.4°	222	20.003	0.677
13.14°	114	14.336	0.674
21.9°	42	8.699	0.675
27.8°	78	11.854	0.675
60°	6	3.289	0.650

**Figure A1:**
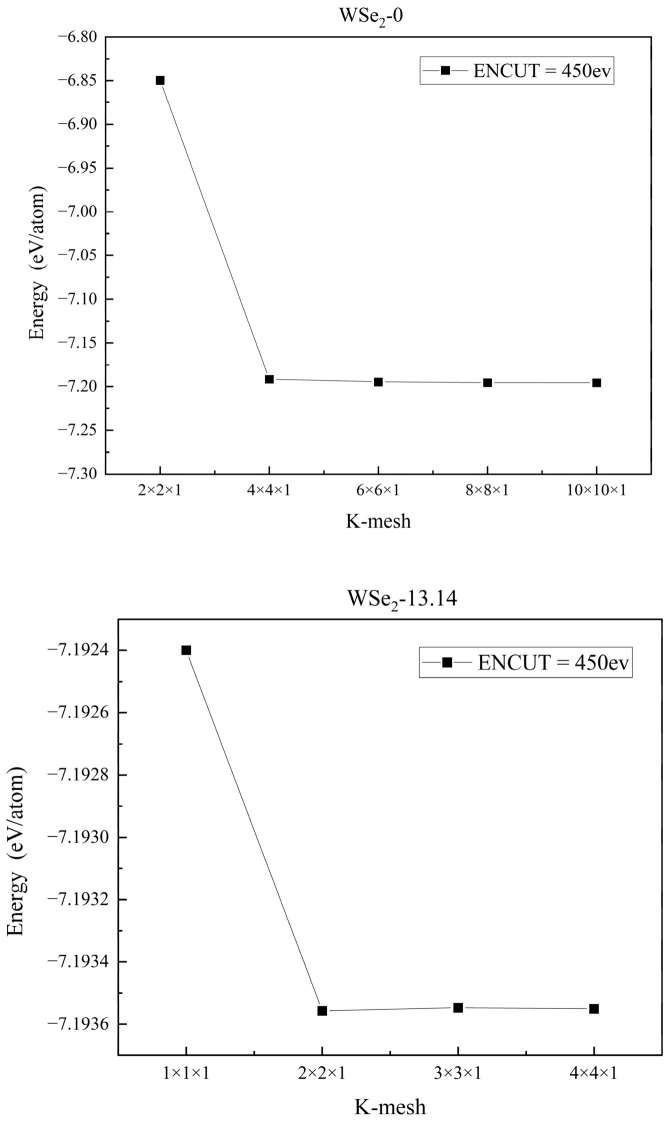

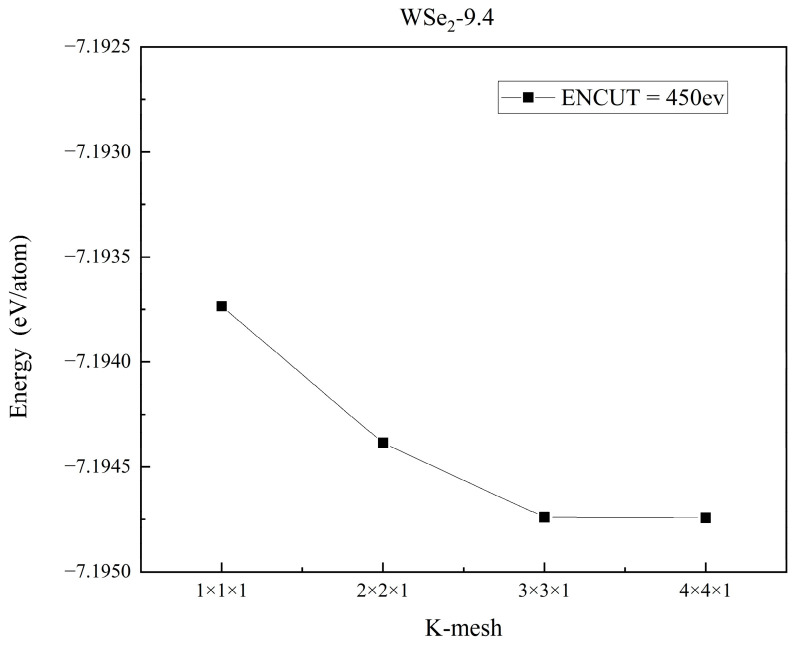
K-mesh convergence test.

## Data Availability

The data presented in this study are contained within the article and the Appendix A and Appendix B.

## Appendix A. K-Mesh Convergence Test

Because the twisted bilayer WSe_2_ systems investigated in this work span a wide range of structural scales, from small primitive cells to large moiré supercells, with correspondingly large variations in the number of atoms, representative k-point convergence tests were carried out to ensure sufficient Brillouin-zone sampling accuracy. The k-point convergence results are shown in Figure A1. Specifically, three typical configurations with twist angles of 0°, 13.14°, and 9.4° were selected, corresponding to relatively small, intermediate, and large supercell sizes, respectively. These models can therefore provide a reasonably comprehensive assessment of the sensitivity of systems with different sizes to the k-point density. The test results show that, with progressive refinement of the k-point mesh, the variations in key physical quantities, including the total energy and band gap, gradually decrease and eventually become negligible, indicating that the adopted k-point settings are sufficiently converged. These results confirm that the k-point meshes employed for different twist-angle configurations in this work achieve a good balance between computational accuracy and cost, thereby ensuring the reliability of the calculated electronic structures and optical properties.

## Appendix B. Orbital Density of States

Consistent with the conclusions drawn from Figure 5, the orbital-projected density-of-states results shown in Figure A2a–f further clarify the orbital origins of the band-edge states in all twisted bilayer WSe_2_ configurations. In the valence-band region (E < 0), states near the band edge close to 0 eV are predominantly contributed by Se p orbitals, which exhibit stable and pronounced intensity, accompanied by noticeable W d contributions. This indicates that the valence-band maximum mainly originates from bonding states formed through hybridization between Se p and W d orbitals. Such behavior is consistent with the well-established picture in WSe_2_, where the valence band is primarily governed by hybridization between chalcogen p states and transition-metal d states. In contrast, W s and Se s states mainly appear at deeper energy levels far below the band edge. In the conduction-band region (E > 0), states close to the conduction-band minimum are dominated by W d orbitals, while Se p orbitals contribute only secondary weight, indicating that the conduction-band edge mainly arises from interlayer-coupled W d states. Therefore, the conclusion derived from Figure 5—that the conduction band is mainly W-derived while the valence band is primarily Se-derived—is further confirmed at the orbital-resolved level. Moreover, for intermediate twist angles (9.4°, 13.14°, 21.9°, and 27.8°), pronounced sharp peaks and identifiable peak reconstructions near the band edges are again observed, reflecting variations in interlayer coupling strength and demonstrating that moiré-induced band reconstruction remains significant under twist modulation.
